# Noninvasive imaging of the thirteen-lined ground squirrel photoreceptor mosaic

**DOI:** 10.1017/S0952523815000346

**Published:** 2016-01-26

**Authors:** BENJAMIN SAJDAK, YUSUFU N. SULAI, CHRISTOPHER S. LANGLO, GABRIEL LUNA, STEVEN K. FISHER, DANA K. MERRIMAN, ALFREDO DUBRA

**Affiliations:** 1 Department of Cell Biology, Neurobiology, & Anatomy, Medical College of Wisconsin, Milwaukee, Wisconsin; 2 Department of Ophthalmology, Medical College of Wisconsin, Milwaukee, Wisconsin; 3 Neuroscience Research Institute, University of California, Santa Barbara, Santa Barbara, California; 4 Department of Biology, University of Wisconsin Oshkosh, Oshkosh, Wisconsin; 5 Department of Biomedical Engineering, Marquette University, Milwaukee, Wisconsin

**Keywords:** Photoreceptors, Adaptive optics, Visual streak, Rodent, Retinal imaging

## Abstract

Ground squirrels are an increasingly important model for studying visual processing, retinal circuitry, and cone photoreceptor function. Here, we demonstrate that the photoreceptor mosaic can be longitudinally imaged noninvasively in the 13-lined ground squirrel (*Ictidomys tridecemlineatus)* using confocal and nonconfocal split-detection adaptive optics scanning ophthalmoscopy using 790 nm light. Photoreceptor density, spacing, and Voronoi analysis are consistent with that of the human cone mosaic. The high imaging success rate and consistent image quality in this study reinforce the ground squirrel as a practical model to aid drug discovery and testing through longitudinal imaging on the cellular scale.

## Introduction

**Fig. 1.**
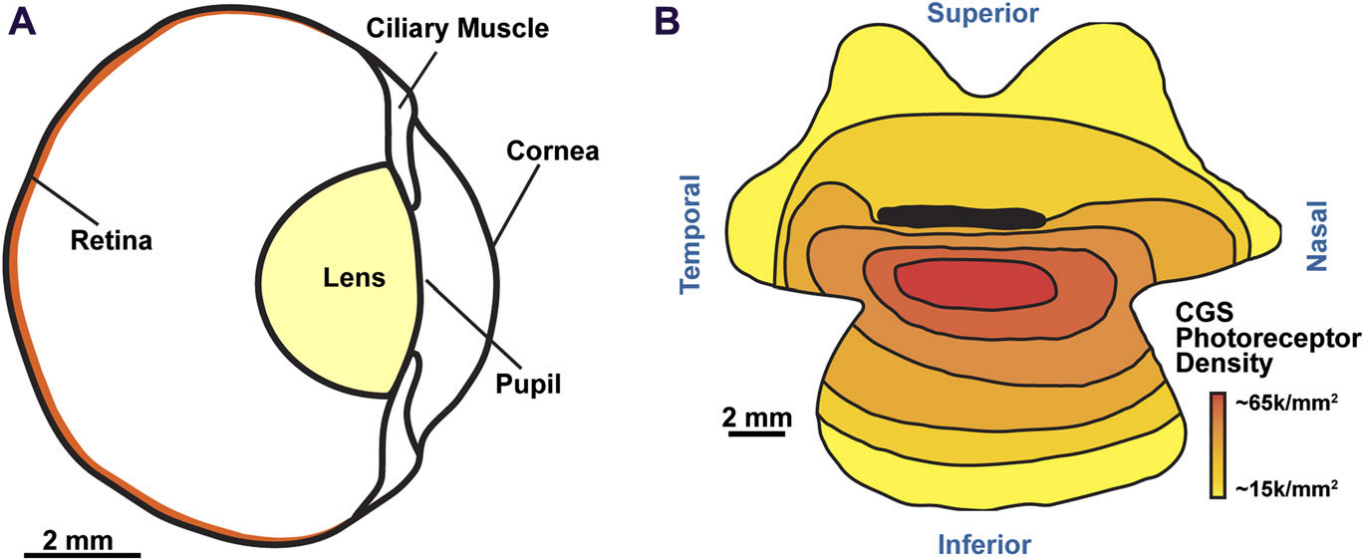
A cross-sectional schematic of 13LGS ocular anatomy (**A**). The horizontal optic nerve head (ONH) lies approximately where “retina” is indicated. Reproduced from Chou & Cullen (1984) and Sussman et al. (2011) with permission. Schematic of photoreceptor density in relation to the horizontal ONH (dark black line) of the California ground squirrel (**B**). The red area of highest cone density denotes the visual streak. Reproduced from Long & Fisher (1983).

Ground squirrels are important animals for investigating vision. Many studies of visual processing, function, and injury have relied upon several species of this cone-dominant, diurnal rodent as a model for the human visual system (for review, see Van Hooser & Nelson, 2006). Ground squirrels are dichromats (Jacobs & Yolton, 1969; Ahnelt, 1985; DeVries & Li, 2004) with medium-wavelength cones with peak sensitivity around 530 nm, and slightly larger short-wavelength cones that make up about 6% of the total (comparable to humans) and have peak sensitivity around 465 nm (Eigner et al., 1984; Kryger et al., 1998). About 86% of photoreceptors in the California ground squirrel are cones, while the remaining 14% of photoreceptors are rods (Jacobs et al., 1980; Kryger et al., 1998). Photoreceptor densities in the this species have been reported to range from 26,000 to 68,200 cells/mm^2^ (Fig. 1B; Long & Fisher, 1983) with a peak cone density in a visual streak about 2 mm inferior from the horizontal optic nerve, and highest rod density in the inferotemporal retina (Kryger et al., 1998).

While early photoreceptor topography studies used histological approaches (Long & Fisher, 1983; Curcio et al., 1990; Kryger et al., 1998), the adaptive optics (AO) fundus camera (Liang et al., 1997) and the AO scanning light ophthalmoscope (AOSLO) (Roorda et al., 2002; Rossi et al., 2011) allow noninvasive visualization of the photoreceptor mosaic. Although the vast majority of studies using this technology to date have been in human subjects, AO ophthalmoscopy has considerable potential for improving our understanding of natural history of disease, as well as aid in the testing of therapeutics through noninvasive longitudinal imaging in animal models.

AO ophthalmoscopes have been used to image feline (Rosolen et al., 2010), chick (Kisilak et al., 2012; Walker et al., 2015), rat (Geng et al., 2007; Geng et al., 2009), and mouse retina (Biss et al., 2007; Geng et al., 2012; Schallek et al., 2013), despite some technical challenges described by Zhou et al. (2012). Impressive strides have been made using noninvasive two-photon imaging of the macaque photoreceptor mosaic (Hunter et al., 2010) and mouse retina (Sharma et al., 2013), as well as functional AO imaging of light responses of ganglion cells in mice (Yin et al., 2013) and macaque (Yin et al., 2014). More recently, Guevara-Torres and colleagues used split-detection, a method previously shown to reveal the cone inner segment mosaic in humans (Scoles et al., 2014), to provide images of mouse photoreceptor distal processes, photoreceptor somata, and horizontal cells (Guevara-Torres et al., 2015). Zawadzki and colleagues combined AOSLO with phase-variance optical coherence tomography and SLO for visualization of cone photoreceptors and microglia expressing enhanced green fluorescent protein in the mouse retina (Zawadzki et al., 2015).

The 13-lined ground squirrel (13LGS) possesses a small lens relative to its eye size (Fig. 1A; Chou & Cullen, 1984; Sussman et al., 2011), similar to the human eye. We believe that this is beneficial for AO retinal imaging, as the optical surfaces that are likely the source of the eye’s monochromatic aberration are close to the exit pupil plane, and thus more amenable to correction with a single wavefront corrector that is optically conjugate to it.

Here, we used a modified custom AOSLO (Dubra & Sulai, 2011; Sulai & Dubra, 2014) capable of simultaneous confocal (reflectance), dark-field (Scoles et al., 2013) and nonconfocal split-detection (Scoles et al., 2014) imaging to visualize the 13LGS photoreceptor mosaic. We then measured photoreceptor density (Chui et al., 2008), spacing (Rossi & Roorda, 2010) and performed Voronoi analysis, all of which were then compared to histological reports in similar species.

## Materials and methods

### Animals

13LGS (*Ictidomys tridecemlineatus*) were obtained from the colony at the University of Wisconsin Oshkosh (Merriman et al., 2012). From January through April, 2015, five nonhibernating 13LGS were housed at the Medical College of Wisconsin under conditions outlined in Merriman et al. (2012), including a naturally lengthening light cycle. Two squirrels in this study were wild-caught adult females of indeterminate age (WC1440 and WC1442), and three (140301, 140402, and 141301) were captive-bred weanlings born in May, 2014 (two males, one female). All experimental procedures were approved by the Institutional Animal Care and Use Committee of the Medical College of Wisconsin.

Squirrels were anesthetized with inhaled isoflurane (5% induction, 2–3% maintenance) in oxygen and placed on a heated rodent alignment stage with two rotational and three translational degrees of freedom. During induction of anesthesia, the pupils were dilated with one drop each of 2.5% phenylephrine and 1% tropicamide. An ocular speculum was used to keep the eyelids open and saline drops were applied as needed to maintain corneal hydration. Respiratory rate and heart rate were continuously monitored during anesthesia, and a warming pad was used to maintain body temperature during anesthesia and recovery.

### Adaptive optics scanning light ophthalmoscopy

**Fig. 2.**
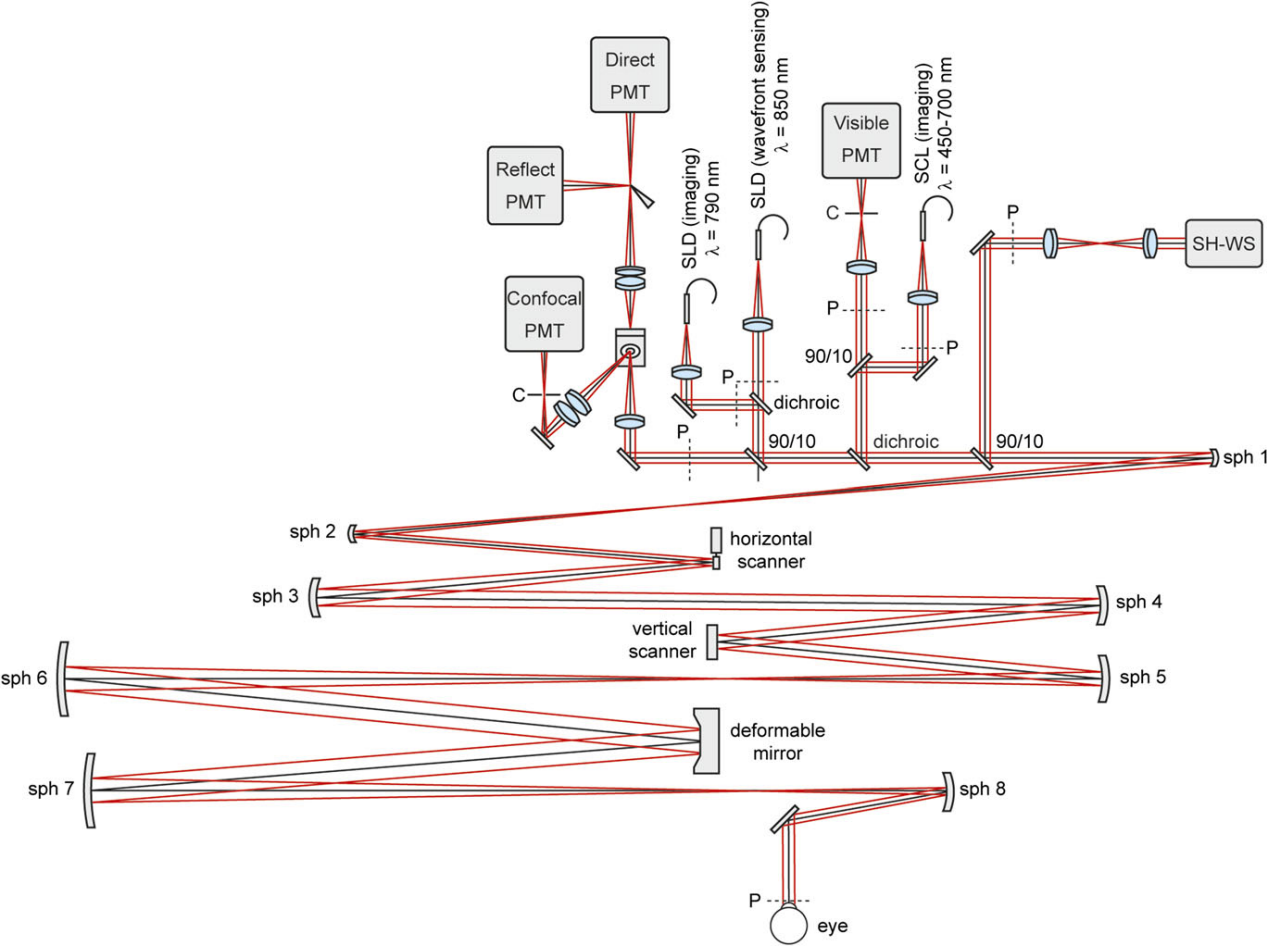
Schematic of the AOSLO modified for ground squirrel imaging. PMT stands for photomultiplier tube, C for confocal pinhole, SLD for superluminescent diode, SCL for super continuum light source, SH-WS for Shack-Hartmann wavefront sensor, sph for spherical mirror, and P indicates planes optically conjugate with the pupil of the eye.

Confocal and nonconfocal reflectance images were acquired using a previously described custom AOSLO modified for a 4 mm pupil diameter (Sulai & Dubra, 2014) (Fig. 2). Illumination was achieved with superluminescent diodes (SLD; Superlum, Carrigtwohill, County Cork, Ireland) for imaging at 790 nm and wavefront sensing at 850 nm. The optical powers measured at the pupil of the eye were 355 *µ*W at 790 nm and 48 *µ*W at 850 nm. Measurement of the eye's wavefront aberrations was performed using a custom Shack–Hartmann wavefront sensor (Dubra & Sulai, 2011), and wavefront correction was achieved with a 7.2 mm diameter 97-actuator deformable mirror (Alpao, Montbonnot, France).

### Photoreceptor mosaic image processing and analysis

After manually selecting a reference image for each recorded sequence, the 20–50 images with highest normalized cross-correlation values were averaged (Dubra & Harvey, 2010). The resulting averages with high signal-to-noise ratio were then manually stitched together using Adobe Photoshop (San Jose, CA). Because the center of the large cone-dominant visual streak is difficult to pinpoint in ground squirrels (Fig. 1B), retinal locations are described relative to superior or inferior distance from the optic nerve head (ONH). Vertical montages of registered images starting from the ONH were used to measure the vertical location, but the horizontal position was not noted because photoreceptor density is known to be uniform along the horizontal meridian (Long & Fisher, 1983). Regions of interests (ROIs) of 80 × 80 *µ*m were cropped from 4 to 8 areas at known distances from the ONH in each squirrel.

An automated cell counting algorithm was used to identify photoreceptors (Li & Roorda, 2007), with subsequent manual correction to identify missed or mislabeled cells. The pixel size in micrometers was calculated using a Ronchi ruling placed in the back focal plane of a 19 mm focal length model eye, and then scaled linearly to the estimated 5 mm focal length of the ground squirrel eye (McCourt & Jacobs, 1984). Photoreceptor density and intercell spacing were calculated for all ROIs using the estimated photoreceptor locations. Packing geometry was measured using previously described Voronoi analysis (Baraas et al., 2007; Carroll et al., 2009).

### Retinal whole-mount immunocytochemistry

Squirrel 140301 was euthanized with an overdose of pentobarbital sodium (120 mg/kg) in August, 2015, and the eyes were prepared for retinal whole-mounts using a previously described protocol (Sakai et al., 2003). In brief, the eyes were enucleated and immersion fixed for 10 min in 4% paraformaldehyde in phosphate-buffered saline (PBS) at pH 7.4. The cornea and lens were then removed and the eyecups were again fixed for 3 days, after which samples were rinsed and stored in PBS. Retinas were separated from the underlying retinal-pigmented epithelium and then further rinsed in PBS. Retinal whole-mounts were initially blocked in Normal Donkey Serum in PBS containing 0.5% bovine serum albumin, 0.1% Triton X-100, 0.1% sodium azide (PBTA) at pH 7.4 (1:20, Jackson Immunoresearch, West Grove, PA) overnight at 4**°**C. Whole-mounts were then placed in PBTA with primary antibodies against M-cone opsin (1:500, EMD Millipore, Billerica, MA) and S-cone opsin (1:100, Santa Cruz Biotechnologies, Santa Cruz, CA) overnight at 4**°**C. Subsequently, primary antibodies were rinsed 3 × 15 min and 1 × 1 h in cold PBTA. Corresponding secondary antibodies donkey antirabbit 488 and donkey antigoat 647 fluorochromes (Jackson Immunoresearch, West Grove, PA) were added for a final overnight incubation at 4**°**C. Samples were then rinsed in 4°C PBTA 3 × 15 min and 1 × 1 h prior to being mounted in 5% *n*-propyl gallate in glycerol on glass slides, covered with a coverslip (#0 thickness, Electron Microscopy Sciences, Hatfield, PA), and sealed with nail polish.

### Image acquisition and registration

Specimens were viewed and images collected using an Olympus FluoView 1000 laser scanning confocal microscope (Center Valley, PA) equipped with an UPlanFLN 40× oil immersion lens, numerical aperture 1.30 as well as a motorized stage (Applied Scientific Instrumentation, Eugene, OR). Optical sections were collected at 1-*µ*m intervals to create individual z-stacks and used to generate maximum intensity projections. Images were collected with a 20% overlap in the *x*–*y* axis to aid montaging. Each z-stack was then registered using the image analysis software Imago version 1.5 (Mayachitra Inc., Santa Barbara, CA). ROIs of 80 × 80 *µ*m were cropped from 9 areas of known distance inferior from the ONH. Photoreceptor density, photoreceptor spacing, and Voronoi analysis were calculated as indicated above.

## Results

### Photoreceptor mosaic imaging

**Fig. 3.**
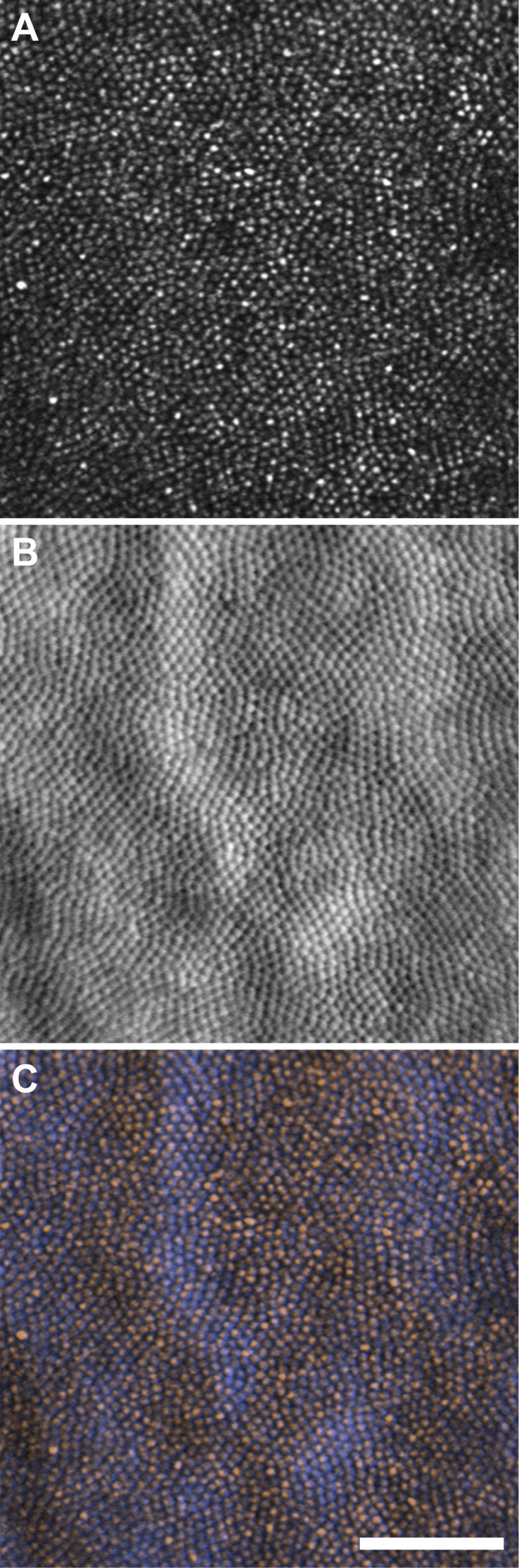
*In vivo* images of the 13LGS photoreceptor mosaic within the visual streak. Confocal (**A**) and split-detector (**B**) images taken from the same location. Color-merged image (**C**), where the confocal image is orange, and split-detector is blue. Note that each blue mound has a corresponding orange spot thought to be a waveguiding outer segment. Scale bar = 50 *µ*m.

**Fig. 4.**
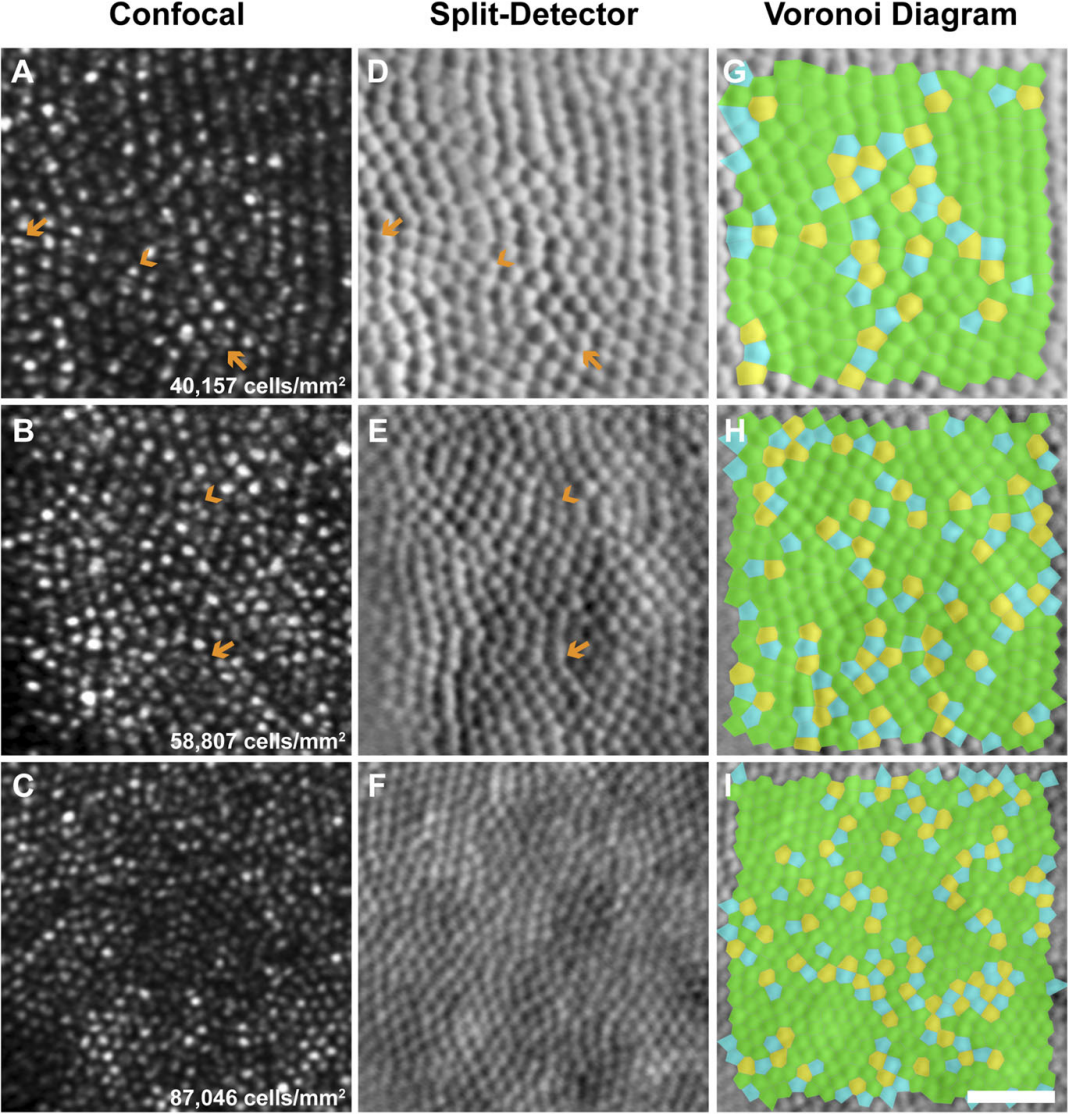
Photoreceptor geometry in the 13LGS (squirrel WC1440). Confocal images (**A**–**C**), split-detector images (**D**–**F**), and the corresponding Voronoi diagrams (**G**–**I**) at three retinal locations. (**A**, **D**, and **G**): Immediately superior of the ONH. (**B**, **E**, and **H**): 0.25 mm inferior of the ONH. (**C**, **F**, and **I**): 2.5 mm inferior of the ONH (i.e., in visual streak). Note cone enlargement with proximity to the ONH (e.g., **D** *vs*. **F**) and high cone density within the visual streak (**C**, **F**, and **I**). The confocal images reveal several multimodal cone outer segments, and the split-detector image reveals a single corresponding inner segment (orange arrows). Rods can be estimated according to their smaller size relative to cones (orange arrowheads). Blue = five-sided, green = six-sided, and yellow = seven-sided. Scale bar = 20 *µ*m.

Simultaneous capture of confocal and split-detection AOSLO images revealed the photoreceptor outer segments as bright spots and the corresponding inner segments as mound-like structures, respectively (Fig. 3). The intensity profile and contrast of these features, as well as their packing geometry remarkably resemble those observed in humans (Scoles et al., 2014). Fig. 4 shows representative images of multiple locations with varying photoreceptor densities, and their corresponding Voronoi diagrams. The split-detector images were used for analysis because the multimodal appearance seen in some photoreceptors with confocal AOSLO made it difficult to determine the cell's center, most notably near the ONH (Fig. 4A and 4B). As many as four bright spots were visualized in some photoreceptors with a single corresponding inner segment (Fig. 4A and 4D).

### Photoreceptor topography

**Fig. 5.**
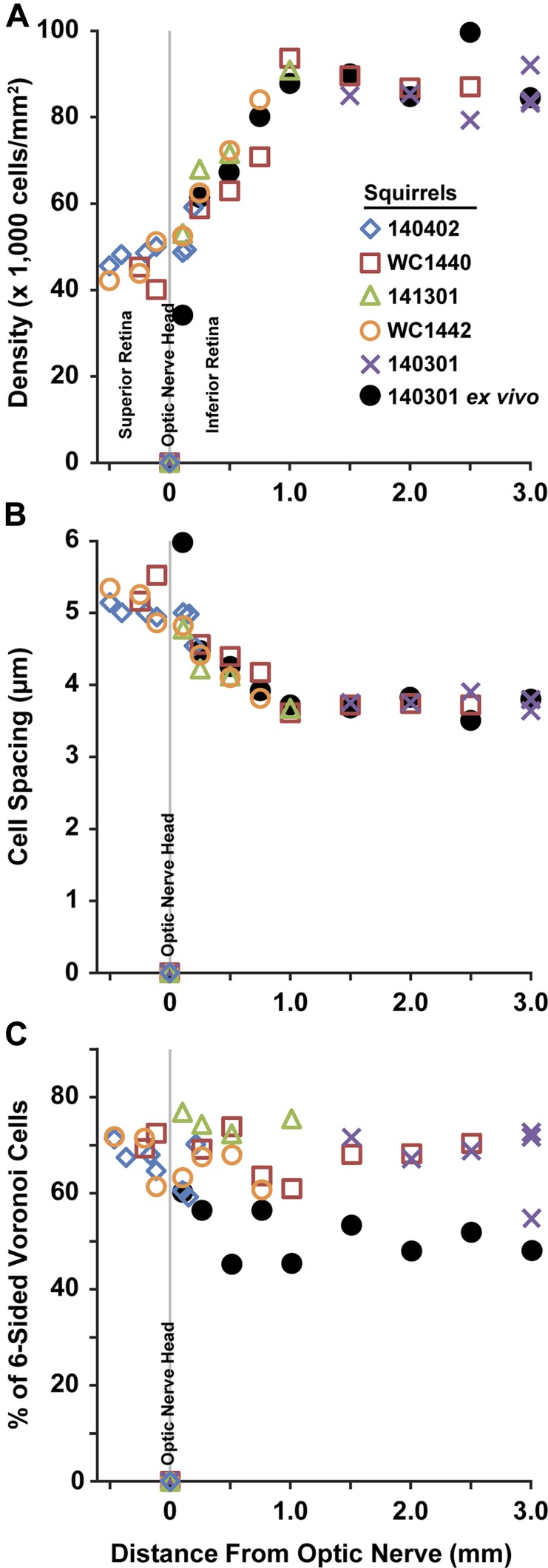
Photoreceptor cell density (**A**), spacing (**B**), and Voronoi cell sidedness (**C**) relative to the ONH in five living 13LGS, and one ex vivo whole-mount.

Imaging at several superior–inferior eccentricities relative to the ONH revealed peak photoreceptor density in the visual streak ranging from 84,025 to 93,668 cells/mm^2^ (*n* = 4). The lowest cell density was found adjacent to the ONH, ranging from 40,157 to 53,118 cells/mm^2^ (*n* = 4). Density values at other locations agreed with the previously described photoreceptor topography as illustrated in Fig. 5A. Previous *ex vivo* whole-mount studies (Long & Fisher, 1983; Kryger et al., 1998) revealed the lowest photoreceptor density in the far periphery of the California ground squirrel retina, adjacent to the ora serrata, a region inaccessible to our AOSLO and most other ophthalmoscopes.

### Photoreceptor mosaic geometry

Average intercell spacing ranged from 3.61 to 5.34 *µ*m (Fig. 5B). Photoreceptors were packed with a triangular geometry with an average of 68% of cells having six-sided Voronoi regions (33 ROIs, 5 animals). This packing geometry was consistent across different retinal locations examined (Figs. 4G–4I and 5C).

### Photoreceptor mosaic histology

**Fig. 6.**
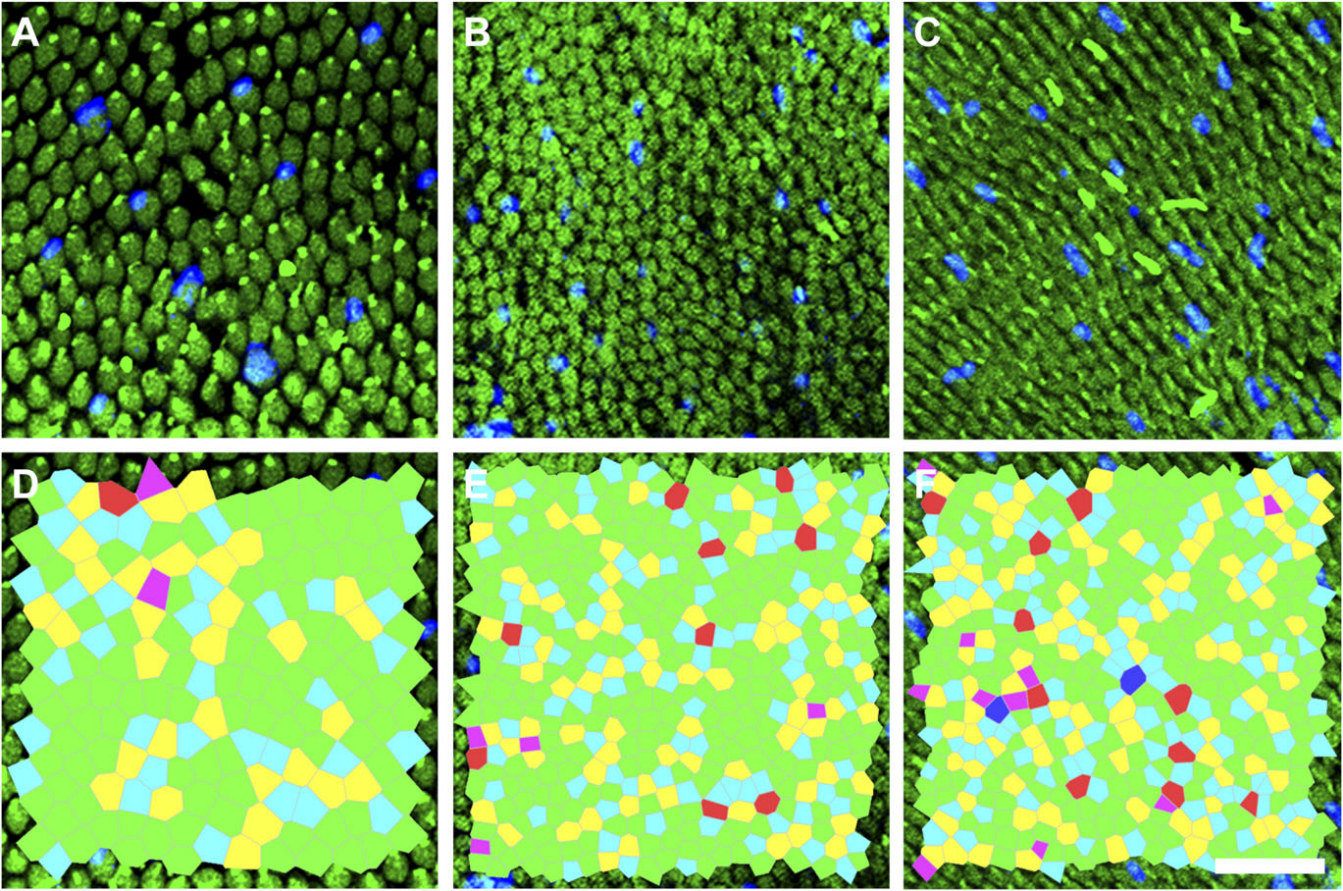
*Ex vivo* single-photon confocal fluorescence microscopy of 13LGS immunostained retinal whole-mount at three locations: Immediately inferior of the ONH (**A** and **D**), 0.750 mm inferior (**B** and **E**), 2 mm inferior (**C** and **F**). Green = M-opsin, and blue = S-opsin (**A**–**C**). Corresponding Voronoi cell overlays (**D**–**F**, magenta = four-sided, blue = five-sided, green = six-sided, yellow = seven-sided, red = eight-sided, and dark-blue = nine-sided). Scale bar = 20 *µ*m.

Retinal whole-mount staining in one 13LGS revealed values consistent with those obtained with AOSLO imaging. Photoreceptor density in the visual streak ranging from 84,340 to 99,638 cells/mm^2^, with the lowest cell density of 33,394 cells/mm^2^, adjacent to the ONH (Fig. 5A). Average photoreceptor spacing ranged from 3.51 to 6.08 *µ*m (Fig. 5B). The packing geometry was less consistent across these nine ROIs, with 45–60% of cells having six-sided Voronoi regions (Fig. 5C). Fig. 6 shows *ex vivo* retinal whole-mount ROIs from three locations, and their corresponding Voronoi cells.

## Discussion

Ground squirrels, including the species studied here, are increasingly being used for studies of visual anatomy and retinal circuitry because they are diurnal rodents, relying overwhelmingly on cone-mediated vision as a primary sense for survival. Here we apply noninvasive AOSLO to this cone-dominant mammal, and demonstrate the practicality of the 13LGS in studying living cone photoreceptors.

The photoreceptor mosaic metrics in this study are consistent with previous topographical reports in the California ground squirrel (Long & Fisher, 1983; Kryger et al., 1998), with lower photoreceptor densities around the ONH and a sharp rise in density inferior from the ONH. The plateauing of this density around 1.0 mm inferior suggests the start of the cone-dense visual streak (Figs 1A and 5A). Interestingly, the photoreceptor density range that we report using AOSLO on the 13LGS exceeds the range obtained from *ex vivo* whole-mounted California ground squirrel retina (Long & Fisher, 1983; Kryger et al., 1998). These dissimilarities are likely explained by species differences (e.g., the California Ground Squirrel is larger than the 13LGS), because our histological density agrees with our *in vivo* data (Fig. 5A), although more work is needed to validate these measurements. Comparing the same animal’s photoreceptor density *in vivo* and *ex vivo* at the same retinal location, and taking individual animal eye axial length into consideration when scaling the AOSLO images is needed to validate *in vivo* photoreceptor mosaic metrics. Photoreceptor type could potentially be determined by size differences, given that S-cones are slightly larger than M-cones, while rods are slightly smaller (Ahnelt, 1985; von Schantz et al., 1994), but to our knowledge there are no data showing how these relative sizes change across the ground squirrel retina. If the axial length of a given animal was known, a 13LGS schematic eye could be scaled to obtain an approximate focal length, as is customary with human AO ophthalmoscopy (Li et al., 2010). The coarse retinal magnification factor estimated above agrees with previously reported findings in ground squirrel [0.10 mm/deg, (Hughes, 1977)]. Geometrical metrics of the photoreceptor mosaic reported here are consistent with that reported for European ground squirrel (Ahnelt, 1985), showing a hexagonal pattern of predominantly six-sided Voronoi cells when imaged with AOSLO. This pattern was less consistent in the images of flat-mounted retina (Figs. 5C and 6C), probably due to changes in photoreceptor orientation during histological processing.

Ground squirrels have near emmetropic optics (Gur & Sivak, 1979; McCourt & Jacobs, 1984), making this rodent model particularly amenable to AO imaging. While contact lenses and careful regulation of body temperature are critical for delaying lens opacity development in rats and mice during imaging using anesthesia, the 13LGSs imaged in this study did not develop cataracts in imaging sessions that lasted up to an hour. Since 13LGSs are obligate hibernators, their intrinsic tolerance for dehydration may be related to the robustness of corneal and lens tissues under anesthesia. The transparency of the cornea and lens throughout the imaging session allowed for 100% AOSLO imaging success rate and visualization of the photoreceptor mosaic, being only limited by eye drift. During the study, we observed that the depth of anesthesia was critical in reducing eye drift during AOSLO imaging. Each animal had its own “sweet spot” where minimum eye drift could be achieved with an isoflurane flow of 2–3% in oxygen. Finding the correct plane of anesthesia to avoid eye drift has been noted in nonhuman primate AOSLO imaging as well (Gray et al., 2008).

The 13LGS photoreceptors seen in this study show many similarities to human parafoveal photoreceptors (Figs. 3, 4C, and 4F), including resemblance to human cones in both the appearance of reflective signal seen in confocal AOSLO (Roorda et al., 2002), and mound-like structures hypothesized to be inner segments with split-detection AOSLO (Scoles et al., 2014). The Voronoi geometry seen in 13LGS also compares well to cone dominated regions of the human retina, showing mostly six-sided Voronoi cells (Ahnelt, 1985; Curcio & Sloan, 1992). Together, these similarities make the 13LGS an attractive model for studying retinal diseases of cone photoreceptors.

Recently, intravitreal administration of adeno-associated virus encoding green fluorescent protein was used to characterize bipolar cell anatomy in 13LGS retina (Light et al., 2012). Combining such genetic tools with noninvasive imaging will provide new avenues for discerning the squirrel retina’s cone-dominant circuitry, as well as any changes concomitant with hibernation or degeneration.
